# Serogroups, virulence genes and antibiotic resistance in Shiga toxin-producing *Escherichia coli* isolated from diarrheic and non-diarrheic pediatric patients in Iran

**DOI:** 10.1186/1757-4749-5-39

**Published:** 2013-12-11

**Authors:** Hassan Momtaz, Farhad Safarpoor Dehkordi, Mohammad Javad Hosseini, Meysam Sarshar, Maliheh Heidari

**Affiliations:** 1 Department of Microbiology, Shahrekord Branch, Islamic Azad University, P.O. Box 166, Shahrekord, Iran; 2 Young Researchers and Elites Club, Islamic Azad University, Shahrekord Branch, Shahrekord, Iran; 3 Molecular Biology Research Center, Baqiyatallah University of Medical Sciences, Tehran, Iran; 4 Master of Science Microbiology, Flavarjan Branch, Islamic Azad University, Flavarjan, Iran

**Keywords:** Shiga toxin-producing *Escherichia coli*, Diarrhea, Pediatric patients, Iran

## Abstract

**Background:**

From a clinical perspective, it is important to know which serogroups, virulence genes and antibiotic resistance patterns are present in Shiga toxin-producing *Escherichia coli* strains in pediatric patients suffering from diarrheic and non-diarrheic infections. This is the first study in Iran that has comprehensively investigated the Shiga toxin-producing *Escherichia coli* -related infection characteristics in diarrheic and non-diarrheic pediatric patients of 0–60 months of age.

**Methods:**

Two-hundred and twenty four diarrheic and 84 non-diarrheic stool specimens were collected from the Baqiyatallah hospital of Tehran, Iran. The stool samples were cultured immediately and those that were *E. coli*-positive were analyzed for the presence of antibiotic resistance genes and bacterial virulence factors using PCR. Antimicrobial susceptibility testing was performed using disk diffusion method.

**Results:**

One-hundred and fifty four out of 224 (68.75%) diarrheic stools and 31 out of 84 (36.90%) non-diarrheic stools harbored *E. coli*. In addition, children in 13–24 month-old age group had the highest incidence of infection with this bacterium (77.63%). A significant difference was found between the frequency of Attaching and Effacing *Escherichia coli* and Enterohaemorrhagic *Escherichia coli* (*P* =0.045). The genes encoding Shiga toxins and intimin were the most commonly detected virulence factors. Among all serogroups studied, O26 (27.04%) and O111 (18.85%) had the highest incidences in the diarrheic and non-diarrheic patients. The incidence of genes encoding resistance against sulfonamide (*sul1*), gentamicin (*aac*(*3*)-*IV*), trimethoprim (*aadA1*), cephalothin (*blaSHV*) and tetracycline (*tetA*) were 82.78%, 68.03%, 60.65%, 56.55% and 51.63%, respectively. High resistance levels against penicillin (100%), tetracycline (86.88%), gentamicin (62.29%) and streptomycin (54.91%) were observed. Marked seasonality in the serogroup distributions was evident, while STEC infections were more common in summer (*P* =0.041).

**Conclusions:**

Our findings should raise awareness about antibiotic resistance in diarrheic pediatric patients in Iran. Clinicians should exercise caution when prescribing antibiotics, especially during the warmer months of the year.

## Background

Infection with Shiga toxin (Stx)-producing *Escherichia coli* (STEC) can result in a spectrum of outcomes, ranging from asymptomatic carriage to uncomplicated diarrhea, hemolytic uremic syndrome (HUS), bloody diarrhea, hemolytic anemia, thrombocytopenia, and acute renal failure [1-4]. High mortality and morbidity rates have been reported for HUS, which can occur from infection with STEC strains [1,5]. The pathogenesis of STEC is related to several bacterial virulence factors [4,6]. Some of the most important of these virulence factors include the intimin (*eae*) protein, two shiga toxins called *stx1* and *stx2*, and the plasmid-encoded protein known as hemolysin (*ehly*) [4,6].

Most outbreaks and sporadic cases of bloody and non-bloody diarrhea and HUS have been attributed to strains of the STEC serogroup O157 [7,8]. However, non-O157 strains such as O26, O111, O145, O26, O91, O103, O113, O128, O121 and O45 have been shown to cause bloody and non-bloody diarrhea and HUS [7,8]. If diarrheic patients do not receive effective treatment, they are susceptible to secondary infections and illnesses.

Diseases caused by *E. coli* often require antimicrobial therapy; however, antibiotic-resistant strains of this bacterium cause longer and more severe illnesses than their antibiotic-susceptible counterparts. Several studies have shown that antibiotic resistance in *E. coli* has increased over time [8-10]. In keeping with this, an epidemiological investigation in Iran revealed that STEC strains were the most commonly detected strains in pediatric patients with diarrhea and that there was a high incidence of resistance (85–100%) to commonly used antibiotics [11,12]. Antibiotic resistance genes are known to cause antibiotic resistance in STEC strains isolated from diarrheic patients [13].

Data on the distribution of serogroups, virulence genes and the antimicrobial resistance properties of STEC strains isolated from pediatric patients are scarce in Iran [11]. Therefore, the aim of the present study was to characterize STEC strains isolated from Iranian diarrheic and non-diarrheic pediatric patients at the molecule level and investigate their susceptibility to 12 commonly used antibiotics, as well as investigating seasonal variation in the prevalence and serogroups distribution of *E. coli*.

## Methods

### Sample collection, preparation, and identification of *E. coli* serogroups

From March 2012 to March 2013, a period covering seasonal variation, 308 stool samples from diarrheic and non-diarrheic pediatric patients were collected from the Baqiyatallah hospital in Tehran, Iran. Stool samples were classified as either diarrheic or non-diarrheic. Individuals from the diarrheic group were placed into six groups based on their ages (1–12, 13–24, 25–36, 37–48 and 49–60 month-old children) (Table 1). Information on the clinical and epidemiological history of these patients was obtained through questionnaires. Patients presented at the hospital with symptoms such as nausea and fever, while others had dysentery-like or inconsequential symptoms. Stool specimens were collected using sterile rectal swabs, which were transferred to tubes containing Stuart medium (Merck, Germany). Samples were transferred to the Biotechnology and Microbiology Research Center of the Islamic Azad University of Shahrekord at 4°C. All samples were diluted in phosphate buffered saline (PBS, Merck, Germany). The samples were plated onto MacConkey’s agar (MC, Merck, Germany) and incubated overnight at 37°C. A lactose positive colony was selected from each sample, streaked onto Eosin Methylene Blue (EMB, Merck, Germany) plates and incubated overnight at 37°C. Green colonies with a metallic luster were considered as typical *E. coli* colonies. Such colonies were confirmed as *E. coli* using standard biochemical tests (e.g., Methyl red, Voges-Proskauer, Indole, and Citrate utilization tests). Colonies were confirmed as *E. coli* by PCR [14]. *E. coli* isolates were stored in Tryptic Soy Broth (TSB, Merck, Germany) containing 20% glycerol at -70°C for further characterization.

**Table 1:** **Incidence of *E. coli *isolated from diarrheic and non**-**diarrheic children**

**Age group (Months)**	**No. samples**	**No. positive samples**
**1**–**12**	52	36 (69.23%)
**13**–**24**	76	59 (77.63%)
**25**–**36**	39	24 (61.53%)
**37**–**48**	24	16 (66.66%)
**49**–**60**	33	19 (57.57%)
**Total diarrheic samples**	224	154 (68.75)
**Non diarrheic**	84	31 (36.90%)
**Total**	308	185 (60.06%)

### Antimicrobial susceptibility testing

Antimicrobial susceptibility testing of the isolates was performed using the Kirby–Bauer disc diffusion method and Mueller–Hinton agar (Merck, Germany) according to Clinical and Laboratory Standards Institute (CLSI) instructions [15]. Inoculated plates were incubated aerobically at 37°C for 18–24 h, after which antimicrobial susceptibility in the *E. coli* isolates were tested. Tetracycline (30 μg/disk), ampicillin (10 u/disk), penicillin (10 u/disk), sulfamethoxazole (25 μg/disk), streptomycin (10 μg/disk), sulfonamide (100 μg/disk), chloramphenicol (30 μg/disk), gentamicin (10 μg/disk), trimethoprim (5 μg/disk), ciprofloxacin (5 μg/disk), enrofloxacin (5 μg/disk), cephalothin (30 μg/disk), and nitrofurantoin (300 μg/disk) were tested. The results were interpreted in accordance with CLSI criteria [16]. *E. coli* ATCC 25922 was used as quality control for antimicrobial susceptibility determination.

### DNA extraction

Bacterial strains were grown overnight in Trypticase Soy Agar (TSA, Merck, Germany) at 37°C. A single colony was suspended in 100 μL of sterile distilled water. After boiling the suspension for 13 min, the suspension was frozen and centrifuged at 14,000 rpm for 15 min to pellet the cell debris [17]. The supernatant was used as a template for PCR amplification.

### PCR detection of serogroups, virulence factors and antibiotic resistance genes in STEC strains

To detect virulence factors, serogroups, and antibiotic resistance genes in the *E. coli* isolates, different PCR assays were used [16,18-29]. All the PCR products were electrophoresed on 1.5% agarose gels that were strained with ethidium bromide and examined under ultraviolet illumination. *E. coli* O159:H20, O157:K88ac:H19, CAPM 6006, and CAPM 5933 strains were used as positive controls and distilled water was used as a negative control.

### Statistical analyses

The data were analyzed using SPSS (Statistical Package for the Social Sciences) software and *P* values were calculated using Chi-square and Fisher's exact tests to identify statistically significant relationships between the following: patient ages, seasonal variations, patient symptoms and distribution of virulence genes, serogroups and antibiotic resistance properties of the STEC strains isolated from diarrheic and non-diarrheic pediatric patients. A *P* value < 0.05 was considered statistically significant.

### Ethical considerations

The present study was authorized by the ethical committee of the Baqiyatallah hospital of Tehran, Iran and the Microbiology, Biotechnology and Infectious Diseases Center of the Islamic Azad University of Shahrekord Branch, Iran. All patients or their parents signed the written informed consent.

## Results and discussion

All of the diarrheic and non-diarrheic stool samples were examined using culture and PCR techniques. From 308 diarrheic and non-diarrheic stool samples, 185 (60.06%) were positive for *E. coli* (Table 1). In addition, 154 out of 224 diarrheic stool samples (68.75%) and 31 out of 84 non-diarrheic stool samples (36.90%) were positive for *E. coli*. The age distribution of the pediatric patients with regard to infection with *E. coli* is shown in Table 1. We found that the 13–24 month-old patients had the highest incidence of *E. coli* (77.63%), while the 49–60 month-old children had the lowest incidence (57.57%).

The distribution of virulence genes in the *E. coli* subtypes isolated from diarrheic and non-diarrheic pediatric patients is shown in Table 2. We found that s*tx1* and *eaeA* were the most commonly detected virulence genes in stool samples from both groups of children. The Attaching and Effacing *E. coli* (AEEC) subtype was the most commonly detected. The EHEC subtype was only detected in the 13–24, 25–36, and 49–60 month-old patients.

**Table 2:** **Distribution of virulence factors in *E. coli *subtypes**

**Age (Month)**	**Subtype**	**No. Positive samples**	**Virulence gene**
	Non detected	12 (33.66%)	-
	EHEC	-	
			*stx1, eaeA, ehly*: -
	AEEC	24 (66.66%)	
**1–12 (52)**			*stx1*: 18 (75.00%)
			*stx2*: 2 (8.33%)
			*eaeA*: 14 (58.33%)
			*stx1, eaeA*: 15 (62.50%)
			*stx2, eaeA*: 7 (29.16%)
			*stx1, stx2, eaeA*: 2 (8.33%)
	Total	36 (69.23%)	
	Non detected	18 (30.50%)	-
	EHEC	2 (3.38%)	
			*stx1, eaeA, ehly*: 2 (100%)
	AEEC	39 (66.10%)	
**13–24 (76)**			*stx1*: 29 (74.35%)
			*stx2*: 4 (10.25%)
			*eaeA*: 24 (61.53%)
			*stx1, eaeA*: 27 (69.23%)
			*stx2, eaeA*: 8 (20.51%)
			*stx1, stx2, eaeA*: 4 (10.25%)
	Total	59 (77.63%)	
	Non detected	6 (25.00%)	-
	EHEC	1 (4.16%)	
			*stx1, eaeA, ehly*: 1 (100%)
	AEEC	17 (70.83%)	
**25–36 (39)**			*stx1*: 13 (76.47%)
			*stx2*: 2 (11.76%)
			*eaeA*: 12 (70.58%)
			*stx1, eaeA*: 7 (41.17%)
			*stx2, eaeA*: 8 (47.05%)
			*stx1, stx2, eaeA*: 2 (11.76%)
	Total	24 (61.53%)	
	Non detected	4 (25.00%)	-
	EHEC		*stx1, eaeA, ehly*: -
**37–48 (24)**	AEEC	12 (75.00%)	*stx1*: 8 (66.66%)
			*stx2*: 1 (8.33%)
			*eaeA*: 6 (50.00%)
			*stx1, eaeA*: 6 (50.00%)
			*stx2, eaeA*: 5 (41.66%)
			*stx1, stx2, eaeA*: 1 (8.33%)
	Total	16 (66.66%)	
	Non detected	7 (36.84%)	-
	EHEC	3 (15.78%)	
			stx1, eaeA, ehly: 3 (100%)
	AEEC	9 (47.36%)	
**49–60 (33)**			stx1: 7 (77.77%)
			stx2: 1 (11.11%)
			eaeA: 6 (66.66%)
			stx1, eaeA: 4 (44.44%)
			stx2, eaeA: 4 (44.44%)
			stx1, stx2, eaeA: 1 (11.11%)
	Total	19 (57.57%)	
	Non detected	16 (51.61%)	
	EHEC		
			stx1, eaeA, ehly
	AEEC	15 (48.38%)	
**Non diarrheic (84)**			stx1: 12 (80.00%)
			stx2: 2 (13.33%)
			eaeA: 13 (86.66%)
			stx1, eaeA: 7 (46.66%)
			stx2, eaeA: 6 (40.00%)
			stx1, stx2, eaeA: 2 (13.33%)
	Total	31 (36.90%)	

Table 3 shows the incidence of STEC serogroups isolated from diarrheic and non-diarrheic patients. O26 (27.04%) had the highest incidence, followed by O111 (18.85%). We also found that 13–24 month-old patients had the highest incidence of the STEC serogroups. The distribution of antimicrobial resistance genes within the STEC serogroup isolated from diarrheic and non-diarrheic pediatric patients is shown in Table 4. Genes that encode resistance to sulfonamide, gentamicin, trimethoprim, cephalothin and tetracycline antibiotics, i.e., *sul1* (82.78%), *aac*(*3*)-*IV* (68.03%), *aadA1* (60.65%), *blaSHV* (56.55%) and *tetA* (51.63%) were the most common antibiotic resistance genes in the diarrheic and non-diarrheic patients. Interestingly, we found that O26 had the highest frequency of antibiotic resistance genes. Antimicrobial resistance in the STEC serogroups isolated from the diarrheic and non-diarrheic patients is shown in Table 5. STEC strains exhibited the highest level of resistance to penicillin (100%), followed by tetracycline (86.88%), gentamicin (62.29%) and streptomycin (54.91%). Descriptions of the seasonal profiles and clinical signs in diarrheic children for each serogroup are shown in Table 6. Samples that were collected in the summer had the highest incidence of STEC serogroups, while those collected in autumn had the lowest incidence. Children with fever symptom had the highest incidence of STEC serogroups. Distribution of antimicrobial resistance pattern of STEC strains in various clinical samples are shown in Table 7. We found that the STEC strains of dysentery and fever samples had the highest levels of antibiotic resistance. The highest levels of antibiotic resistance of the STEC strains isolated from children with dysentery were found against streptomycin, sulfamethoxazole, gentamicin, cephalothin, nitrofurantoin and ampicillin.

**Table 3:** **Incidence of Shiga toxin**-**producing *E. coli *serogroups**

**Age (Month)**	**O157**	**O26**	**O103**	**O111**	**O145**	**O45**	**O91**	**O113**	**O121**	**O128**
**1**–**12 (24)**	-	7	2	5	2	1	3	2	1	1
**13**–**24 (41)**	2	10	3	7	4	4	5	1	3	2
**25**–**36 (18)**	1	4	2	3	-	1	3	-	4	-
**37**–**48 (12)**	-	4	-	-	2	1	1	-	2	2
**49**–**60 (12)**	3	2	1	2	-	1	-	1	1	1
**Non diarrheic (15)**	-	6	-	6	-	3	-	-	-	-
**Total (122)**	6 (4.91%)	33 (27.04%)	8 (6.55%)	23 (18.85%)	8 (6.55%)	11 (9.01%)	12 (9.83%)	4 (3.27%)	11 (9.01%)	6 (4.91%)

**Table 4:** **Distribution of antimicrobial resistance genes in Shiga toxin**-**producing *E. coli *** **serogroups**

**Serogroups**	***aadA1***	***tetA***	***tetB***	***dfrA1***	***qnr***	***aac (3)- IV***	***sul1***	***blaSHV***	***CITM***	***cat1***	***cmlA***
**O157 (6)**	2	2	2	-	-	2	-	4	2	-	-
**O26 (33)**	25	18	17	12	8	29	31	19	22	1	-
**O103 (8)**	4	8	-	-	-	3	2	5	4	-	-
**O111 (23)**	11	7	10	8	6	16	20	12	11	-	-
**O145 (8)**	4	6	4	6	-	-	8	6	3	-	-
**O45 (11)**	5	7	4	9	-	8	10	8	5	-	-
**O91 (12)**	5	6	5	4	1	11	10	8	6	-	1
**O113 (4)**	4	2	2	-	-	4	4	3	1	-	-
**O121 (11)**	9	6	-	4	-	7	10	4	3	-	-
**O128 (6)**	5	1	3	1	-	3	6	-	2	-	-
**Total (122)**	74 (60.65%)	63 (51.63%)	47 (38.52%)	44 (36.06%)	15 (12.29%)	83 (68.03%)	101 (82.78%)	69 (56.55%)	59 (48.36%)	1 (0.81%)	1 (0.81%)

**Table 5:** **Antimicrobial resistance properties in Shiga toxin**-**producing *E. coli *serogroups**

**Serogroups**	TE30^*^	**S10**	**C30**	**SXT**	**GM10**	**NFX5**	**CF30**	**CIP5**	**TMP5**	**F/M300**	**AM10**	**P10**
**O157 (6)**	4	2	-	-	2	-	3	-	-	-	2	6
**O26 (33)**	32	24	1	29	26	3	17	-	11	1	18	33
**O103 (8)**	8	3	-	1	3	-	4	1	-	-	2	8
**O111 (23)**	16	11	-	19	14	2	11	2	8	-	8	23
**O145 (8)**	8	4	-	8	-	-	4	-	4	1	2	8
**O45 (11)**	10	5	-	11	7	1	7	-	8	-	2	11
**O91 (12)**	10	4	1	8	10	1	6	-	4	-	6	12
**O113 (4)**	4	-	-	3	4	-	2	-	-	-	1	4
**O121 (11)**	9	9	-	10	7	1	2	-	3	-	3	11
**O128 (6)**	5	5	-	5	3	-	-	-	1	-	-	6
**Total (122)**	106 (86.88%)	67 (54.91%)	2 (1.63%)	94 (77.04%)	76 (62.29%)	8 (6.55%)	56 (45.90%)	3 (2.45%)	39 (31.96%)	2 (1.63%)	44 (36.06%)	122 (100%)

*TE30 = tetracycline (30 μg/disk); *S10* = (10 μg/disk); *C*30 = chloramphenicol (30 μg/disk); *SXT* = sulfamethoxazole (25 μg/disk); *GM*10 = gentamycin (10 μg/disk); *NFX*5 = enrofloxacin (5 μg/disk); *CF*30 = cephalothin (30 μg/disk); *CIP*5 = ciprofloxacin (5 μg/disk); *TMP*5 = trimethoprim (5 μg/disk); *F/M300* = nitrofurantoin (300 μg/disk); *AM*10 = ampicillin (10 u/disk); *P*10 = penicillin (10 u/disk).

**Table 6:** Seasonal distribution of serogroups and clinical signs in diarrheic children

**Criteria**	**O157**	**O26**	**O103**	**O111**	**O145**	**O45**	**O91**	**O113**	**O121**	**O128**
	**(6)**	**(27)**	**(8)**	**(17)**	**(8)**	**(8)**	**(12)**	**(4)**	**(11)**	**(6)**
**Spring (32)**	-	9	-	3	5	6	2	-	5	2
**Summer (40)**	4	10	3	5	2	1	7	2	3	3
**(Autumn) (17)**	-	2	3	9	-	1	-	-	1	1
**Winter (18)**	2	6	2	-	1	-	3	2	2	-
**Nausea (42)**	-	7	1	6	5	5	5	3	7	3
**Fever (34)**	2	17	1	8	-	1	3	1	1	-
**Dysentery (14)**	4	3	1	-	2	-	2	-	1	1
**Other clinical signs (17)**	-	-	5	3	1	2	2	-	2	2

**Table 7:** **Distribution of antimicrobial resistance pattern of Shiga toxin producing *Escherichia coli *** **in various clinical samples**

**Clinical signs**	**Antibiotic resistance pattern (%)**											
	**TE30**	**S10**	**C30**	**SXT**	**GM10**	**NFX5**	**CF30**	**CIP5**	**TMP5**	**F/M300**	**AM10**	**P10**
**Nausea (42)**	33 (78.57)	15 (35.71)	-	28 (66.66)	13 (30.95)	1 (2.38)	11 (26.19)	-	9 (21.42)	-	10 (23.80)	42 (100)
**Fever (34)**	34 (100)	22 (64.70)	-	32 (94.11)	27 (79.41)	2 (5.88)	17 (50)	-	10 (29.41)	-	11 (32.35)	34 (100)
**Dysentery (14)**	14 (100)	14 (100)	1 (7.14)	14 (100)	14 (100)	3 (21.42)	12 (85.71)	2 (14.28)	11 (78.57)	2 (14.28)	13 (92.85)	14 (100)
**Other clinical signs (17)**	17 (100)	12 (70.58)	1 (5.88)	16 (94.11)	14 (82.35)	2 (11.76)	13 (76.47)	1 (5.88)	7 (41.17)	-	7 (41.17)	17 (100)
**Non diarrheic (15)**	8 (53.33)	4 (26.66)	-	4 (26.66)	8 (53.33)	-	3 (20)	-	2 (13.33)	-	3 (20)	15 (100)
**Total (122)**	106 (86.88%)	67 (54.91%)	2 (1.63%)	94 (77.04%)	76 (62.29%)	8 (6.55%)	56 (45.90%)	3 (2.45%)	39 (31.96%)	2 (1.63%)	44 (36.06%)	122 (100%)

Our work has identified marked seasonality in the incidence of STEC serogroup strains in diarrheic and non-diarrheic pediatric patients. There were significant differences (*P* =0.041) in the incidence of STEC serogroup strains between the hot and cold seasons of the year. One possible explanation for the high prevalence of STEC serogroups in summer in Iran is that climatic variables such as heat, rain and thunderstorms, together with variable barometric pressure may have affected the patients’ autonomic nervous systems. These variables could affect immunity, thus making people more susceptible to infections. Alternatively, the higher prevalence of STEC serogroups may be related to higher growth rates in the bacteria. Therefore, the highest levels of pediatric health care should be performed during the warmer months of year. Of the studies that have been conducted [30,31] in this field, all have shown a seasonal distribution for *E. coli* with the highest numbers of cases occurring during the warmer months of the year [30,31].

Data from 1996 to 2011 shows a seasonal increase in cases from April to November in Louisiana, USA [30]. Our results show that fever was most commonly associated with the highest incidence of STEC serogroup types (Table 7). This finding is in accordance with the results of Rivas et al. [32].

The most commonly infected group was 13–24 month-old age group (77.63%), but there were no significant differences in the incidence of STEC strains among the various age groups. In addition, 68.75% of diarrheic and 36.90% of non-diarrheic stool samples from the children were positive for *E. coli* strains; hence, the diarrheic patients showed the higher incidence of *E. coli* (*P* = 0.022). Lower incidence of STEC strains in Iranian diarrheic children were reported previously by Salmanzadeh-Ahrabi et al. [33] (15.5% incidence rate in pediatrics under 5 years old) and Alikhani et al. [34] (8.7% incidence rate in pediatrics under 10 years old).

Another important finding relates to the distributions of four bacterial virulence factors (*stx1*, *stx2*, *ehly* and *eaeA*) in the diarrheic and non-diarrheic patients. We found statistically significant (*P* =0.016) association between the incidence of *stx1* and *stx2* genes in all groups and between the EHEC and AEEC subtypes (*P* =0.045). In addition, there were significant differences amongst the incidence of virulence factors in diarrheic and non-diarrheic patients (*P* =0.047). Sang et al. [35] reported that 141 out of 380 diarrheic patients (37.1%) were positive for *E. coli* and of these, ETEC, STEC, EAEC, EIEC and EPEC strains comprised 29.8%, 24.1%, 14.2% 12.8% and 3.5%, respectively. The presence of multiple *stx1*, *eaeA*, and *ehly* genes was found in the EHEC strains isolated from 13–24 (100%), 25–36 (100%) and 49–60 month-old (100%) patients. Additionally, 13.33% of non- diarrheic, 11.11% of 49–60 month-old diarrheic, 8.33% of 37–48 month-old diarrheic, 11.76% of 25–36 month-old diarrheic, 10.25% of 13–24 month-old diarrheic and 8.33% of 1–12 month-old diarrheic patients harbored *stx1*, *stx2* and *eaeA* genes. Similar findings have been reported in various parts of the world including Kenya, Korea, Australia and Brazil [35-38]. Of several studies which were conducted in this field in Iran. Shams et al. [39] showed that 2.8% of STEC isolates were positive for *stx1* or *stx2* genes, Aslani and Bouzari [40] showed that 96.5% of STEC isolates had *stx1* gene, 44.8% had *hly* gene, 3.5% had *stx2*, and 24.1% had *ast1* gene and Bonyadian et al. [41] reported that 27.6% of STEC isolates had *stx1* gene, 20.7% had *hly* gene and 6.9% had *stx2* which all was lower than our results. These studies indicate that the *stx1*, *stx2*, *hly* and *eaeA* genes are only present in some of the groups studied and that the *eaeA* and *ehly* genes are associated with diarrhea. Within the disease-associated strains, those containing *stx1* and *stx2* (especially *stx2*) appear to be more commonly responsible for serious complications bloody and non-bloody diarrhea and HUS than those containing other putative bacterial pathogenic genes [42,43].

O26 was the most commonly detected serogroup of all of the diarrheic and non-diarrheic STEC strains. Statistical analyses showed a significant (*P* =0.028) association between the incidence of the O26 serogroup and other STEC serogroups in all of the study groups. High differences in the incidence of STEC serogroups between diarrheic and non-diarrheic patients was reported previously [44,45]. Aslani and Alikhani [46] reported that O142, O111, O86 and O127 were the most commonly detected serogroups in Iranian diarrheic children under 5 years of age which was in contrast with our results. High presence of O55, O26, O111, O125, O128, O114, O86, O142, O119, O127, and O126 serogroups was reported in the STEC strains of Alikhani et al. investigation [47].

In two studies involving 11 sites in the USA in 1997 and four sites during 1998–1999, screening of 13,798 stool samples for STEC yielded a frequency of 0.9% (120 cases of STEC), and of these 120 cases, from the isolates available, 54% were O157 STEC, while 46% were non-O157 STEC [45]. Many studies have shown that O26 is the most clinically important genotype within the STEC serogroup isolated from diarrheic and even non-diarrheic patients [48,49] because of its association with bloody diarrhea, non-bloody diarrhea and HUS.

Because inappropriate prescriptions of antibiotics causes antibiotic resistance, it was not surprising that our study found that resistance to penicillin was 100%, while resistance to tetracycline, gentamicin and streptomycin were 86.88%, 62.29%, 54.91%, respectively. In terms of bacterial antibiotic resistance genes, *sul1* (82.78%), *aac*(*3*)-*IV* (68.03%), *aadA1* (60.65%), *blaSHV* (56.55%) and *tetA* (51.63%) were the most commonly detected. There were statistically significant differences (*P* =0.029) amongst the incidences of *sul1*, *cat1* and *cmlA* and among *aac*(*3*)-*IV*, *cat1* and *cmlA* (*P* =0.041). Our results showed that the O26 serogroup had the highest incidence of antibiotic resistance genes and resistance to antibiotics. Our results showed that 1.63% of the STEC strains were resistance to chloramphenicol. Chloramphenicol is a banned antibiotic and the slight antibiotic resistance to this drug detected in our study indicates that irregular and unauthorized use of it may have occurred in Iran. Similar chloramphenicol resistance profiles to our own have been reported previously [50,51]. Fazeli and Salehi [52] showed that 72.4%, 65.5% and 58.6% of STEC strains isolated from Iranian diarrheal patients were resistant to amoxicillin, trimethoprim-sulfamethoxazole and tetracycline, respectively.

There were statistically significant differences (*P* =0.040) amongst the incidences of antibiotic resistance in the STEC strains isolated from dysentery, fever, other clinical signs and non-diarrheic samples. The highest incidence of antimicrobial resistance in non-O157 STEC strains [9] was found for sulfisoxazole (36%), tetracycline (32%), streptomycin (29%), ampicillin (10%), trimethoprim (8%), co-trimoxazole (8%), chloramphenicol (7%), kanamycin (7%), piperacillin (6%) and neomycin (5%); these values are lower than the values reported in the present study. Jafari et al. [13] showed that diarrheagenic *E. coli* isolates of Iran had high resistance against amoxicillin and tetracycline (75.5%) which was similar to our results. Similar Iranian investigations have been done previously [52-54]. Käppeli et al. [8] reported that the STEC antibiotic resistance rates against ampicillin, amoxicillin/clavulanic acid, cephalothin, cefpodoxime, cefuroxime, gentamicin, and tetracycline were 3.1%, 12.4%, 1%, 1%, 2.1%, and 21.6%, respectively which was similar to our findings.

Our results showed that resistance to at least one of the 12 antimicrobial drugs tested was identified in all (100%) of the STEC strains, which is consistent with previous studies [8-10]. Our antimicrobial resistance data are in accordance with the results from a study in Spain, in which 238 (41%) of 581 non-O157 STEC strains were resistant to at least one out of the 26 antimicrobial drugs that were tested [9].

The above data highlight large differences in the prevalence of STEC strains in the different studies, as well as differences in virulence genes and antibiotic resistance properties in the clinical samples. This could be related to differences in the type of sample (stool, blood, urine, meat, milk, vegetable and other clinical samples) tested, number of samples, method of sampling, experimental methodology, geographical area, antibiotic prescription preference among clinicians, antibiotic availability, and climate differences in the areas where the samples were collected, which would have differed between each study.

## Conclusions

In conclusion, we identified a large number of virulence factors, serogroups, and antibiotic resistance genes and resistance to more than one antibiotic in the STEC strains isolated from diarrheic and non-diarrheic patients. Our data indicate that O157 and especially non-O157 STEC strains are predominant in Iranian diarrheic children. Marked seasonal variation in the serogroup distribution was also found. Our data revealed that the O26 serogroup, the *stx1*, *stx2*, *eaeA* and *ehly* putative virulence genes, the *sul1*, *aac*(*3*)-*IV*, *aadA1*, *blaSHV* and *tetA* antibiotic resistance genes, and resistance to penicillin, tetracycline, gentamicin and streptomycin were the most commonly detected characteristics of the STEC strains isolated from Iranian diarrheic and non-diarrheic children. Hence, judicious use of antibiotics is required by clinicians.

## Abbreviations

E. coli: *Escherichia coli*; PCR: Polymerase chain reaction; EHEC: Enterohaemorrhagic *Escherichia coli*; AEEC: Attaching and effacing *Escherichia coli*; STEC: Shiga toxin-producing *Escherichia coli*; SPSS: Statistical package for the social sciences; CLSI: Clinical and laboratory standards Institute.

## Competing interests

The authors declare that they have no competing interests.

## Authors’ contributions

DNA extraction, PCR, manuscript preparation, statistical analysis and project support were all performed by HM and FSD. Sample collections and coordination was performed by MJH, MH and MS. All authors have read and approved the final manuscript.
